# Renin–angiotensin system impairs macrophage lipid metabolism to promote age-related macular degeneration in mouse models

**DOI:** 10.1038/s42003-020-01483-2

**Published:** 2020-12-09

**Authors:** Norihiro Nagai, Hirohiko Kawashima, Eriko Toda, Kohei Homma, Hideto Osada, Naymel A. Guzman, Shinsuke Shibata, Yasuo Uchiyama, Hideyuki Okano, Kazuo Tsubota, Yoko Ozawa

**Affiliations:** 1 Laboratory of Retinal Cell Biology, Department of Ophthalmology, Keio University School of Medicine, 35 Shinanomachi, Shinjukuku Tokyo, 160-8582 Japan; 2 Department of Ophthalmology, Keio University School of Medicine, 35 Shinanomachi, Shinjukuku Tokyo, 160-8582 Japan; 3 Department of Physiology, Keio University School of Medicine, 35 Shinanomachi, Shinjukuku Tokyo, 160-8582 Japan; 4 Department of Cellular and Molecular Neuropathology, Juntendo University Graduate School of Medicine, Bunkyo-Ku Tokyo, 113-0033 Japan; 5 Department of Ophthalmology, St. Luke’s International Hospital, 9-1 Akashi-Cho, Chuo-Ku Tokyo, 104-8560 Japan; 6 St. Luke’s International University, 9-1 Akashi-Cho, Tokyo, 104-8560 Japan

**Keywords:** Macular degeneration, Mechanisms of disease

## Abstract

Metabolic syndrome, a condition involving obesity and hypertension, increases the risk of aging-associated diseases such as age-related macular degeneration (AMD). Here, we demonstrated that high-fat diet (HFD)-fed mice accumulated oxidized low-density lipoprotein (ox-LDL) in macrophages through the renin–angiotensin system (RAS). The ox-LDL-loaded macrophages were responsible for visual impairment in HFD mice along with a disorder of the retinal pigment epithelium (RPE), which is required for photoreceptor outer segment renewal. RAS repressed ELAVL1, which reduced PPARγ, impeding ABCA1 induction to levels that are sufficient to excrete overloaded cholesterol within the macrophages. The ox-LDL-loaded macrophages expressed inflammatory cytokines and attacked the RPE. An antihypertensive drug, angiotensin II type 1 receptor (AT1R) blocker, resolved the decompensation of lipid metabolism in the macrophages and reversed the RPE condition and visual function in HFD mice. AT1R signaling could be a future therapeutic target for macrophage-associated aging diseases, such as AMD.

## Abstract (web summary)

Nagai et al. show that mice fed high-fat diet (HFD) accumulate oxidized low-density lipoprotein in macrophages through the renin–angiotensin system, which impairs visual function. They find that angiotensin II type 1 receptor (AT1R) improves the visual function of HFD mice, suggesting AT1R signaling as a potential therapeutic target for age-related macular degeneration.

## Introduction

Metabolic syndrome, a condition involving obesity and hypertension, increases the risk of aging-associated diseases, such as atherosclerotic heart disease^1^, cancers^1^, and age-related macular degeneration (AMD)^2,3^. These diseases are mediated by macrophage-associated inflammation^4–13^ caused by insufficient cholesterol efflux from the macrophages^10,12,13^. However, the molecular mechanisms causing abnormal cholesterol efflux by lipid loading have not been fully understood.

The influence of pathological cholesterol efflux has been analyzed under deficiency of the ATP-binding cassette transporter (ABCA1), a component of the cholesterol efflux system; this deficiency can cause macrophage activation and promote choroidal neovascularization, one aspect of AMD, in mice^10^. AMD is a leading cause of blindness worldwide, and its prevalence is increasing in the current aging society^14–16^. This disease progresses gradually and eventually causes irreversible atrophy of the retina and retinal pigment epithelium (RPE) due to inflammation with or without resulting development of choroidal neovascularization in the neighboring connective tissue, the choroid, at the macular region. In the background, subclinical pathological conditions persist for decades and involve the aging of the RPE. The RPE contributes to visual function by phagocytosing photoreceptor outer segments (OSs), which are composed of lipid bilayers and are rich in visual pigments, and by digesting them in lysosomes to renew the OSs daily^17^. Thus, when the RPE is aged and overworked, lipid deposits comprised of cholesterol accumulate in and around the RPE to gradually form drusen and pseudodrusen^18–22^. The disorganized RPE secretes inflammatory cytokines, such as monocyte chemotactic protein-1 (MCP-1)^23^, to recruit macrophages during the development of choroidal neovascularization, and the progression of AMD in mice^24,25^. In fact, single-nucleotide polymorphisms (SNPs) of cholesterol metabolism-related molecules, such as *ABCA1*^26,27^, and *apolipoprotein E* (*ApoE*); in particular, *APOE4*^28–30^, in addition to high-fat diet (HFD)^31^ and obesity^3,32^, are risk factors for AMD. Thus, cholesterol metabolism and metabolic syndrome are closely related to AMD pathogenesis.

The renin**–**angiotensin system (RAS) is a systemic regulator of blood pressure and body electrolytes^33^, and one of the key regulators of metabolic syndrome^34^. RAS is activated by a series of enzymatic cascades initiated by the substrate, angiotensinogen, which is converted to the active peptide angiotensin II through angiotensin I, and binds to specific receptors, such as the angiotensin II type 1 receptor (AT1R). Angiotensin II produced during systemic circulation is called systemic RAS and causes hypertension through AT1R^35^. AT1R blockers (ARBs) are now widely used as antihypertensive drugs^36^. A set of RAS components are also found in each tissue, which is called tissue RAS, and their effects can be tissue-specific, causing cardiac and renal diseases;^35^ these are also involved in metabolic syndrome^34^. The tissue RAS also regulates neural diseases, such as those in the brain^37^ and the retina^38–42^. AT1R signaling induces retinal synaptic disorders^39^ and inflammation in diabetes^39^ or other diseases^38,40,41^ and affects photoreceptor survival after light exposure^42^.

Given that HFD which often causes metabolic syndrome is related to AMD^43,44^, RAS could be involved in the AMD pathogenesis. Moreover, a clinical study reported that HFD-induced RAS in humans^45^. Taken together, we hypothesized that RAS is involved in AMD pathogenesis and related to impaired lipid metabolism which can cause macrophage activation^10,12,13^. Determining the mechanism of HFD-induced macrophage pathogenesis focusing on RAS may reveal the mechanisms of cholesterol efflux and improve the understanding of the pathogenesis of AMD, as well as other diseases related to metabolic syndrome.

In the current study, we analyze the effects of AT1R blockade in regulating cholesterol efflux and macrophage activation and explore a future therapeutic approach for preventing metabolic syndrome and the associated AMD progression and vision loss in the future.

## Results

### Visual function was impaired in HFD-fed mice and reversed via AT1R blockade

**Fig. 1:**
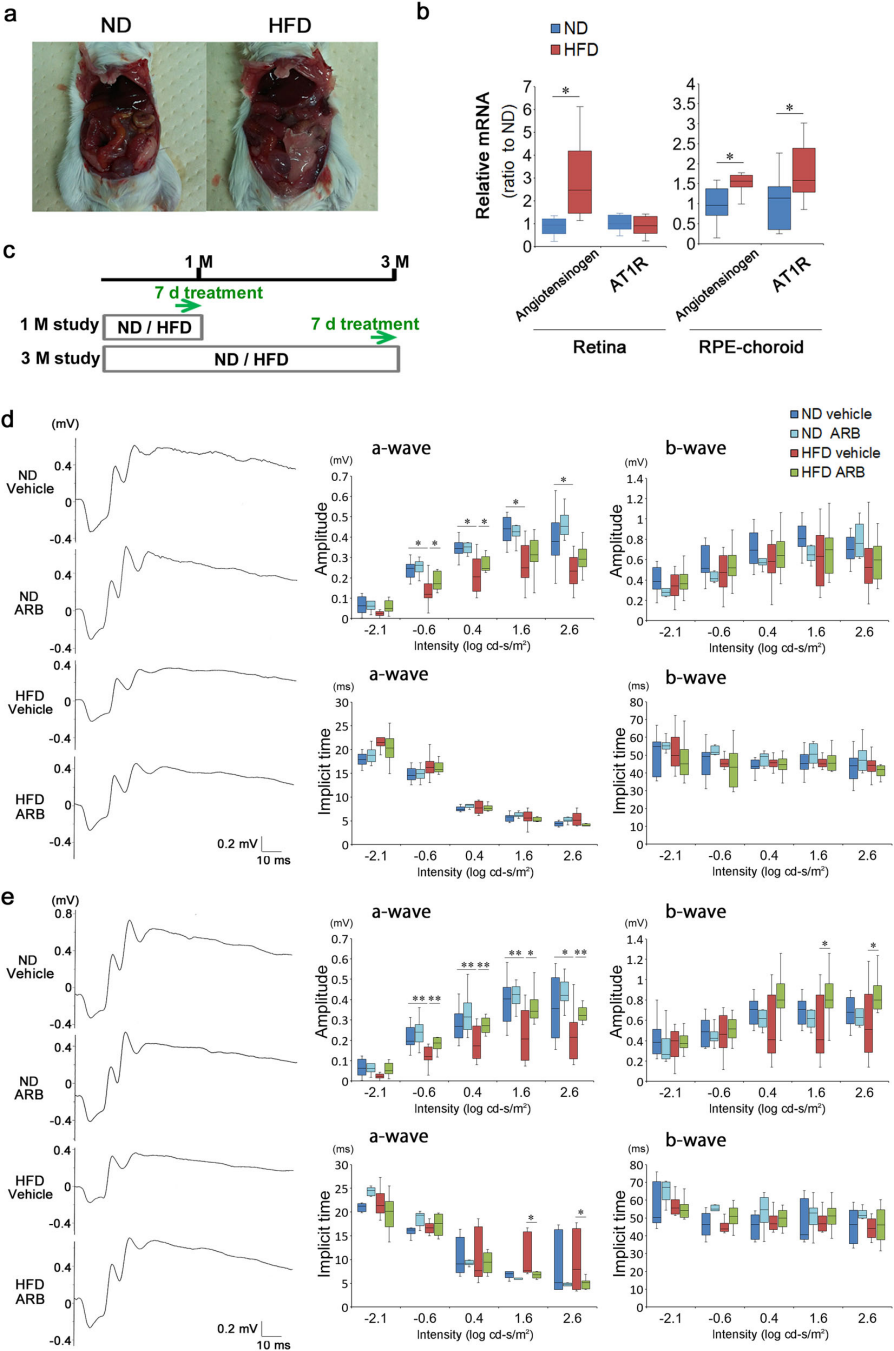
Visual function was impaired in HFD mice, which was then recovered by AT1R blockade. **a**, **b** Regular intake of HFD-induced obesity in BALB/c mice (**a**), and upregulated angiotensinogen in the retina and RPE–choroid tissue, and AT1R in the RPE–choroid tissue (**b**). **c** Intraperitoneal injection of either vehicle or an ARB, valsartan, was performed once per day for the last 7 days of both experiments (1- and 3-month study), to ND- and HFD-fed mice. **d**, **e** Representative ERG waves formed from individual mice, and amplitudes and implicit times of a- and b-waves of each group (**d** at 1 month; **e** at 3 months). Impaired visual function by HFD was recovered by ARB. The a-wave shows photoreceptor function. ND normal diet, HFD high-fat diet, AT1R angiotensin II type 1 receptor, ARB AT1R blocker (valsartan), RPE retinal pigment epithelium, ERG electroretinogram; **b** *n* = 10 for each group. **d**, **e** Respective numbers for ND mice treated with control, ND mice treated with ARB, HFD mice treated with control, and HFD mice treated with ARB were 12, 8, 12, 12. The samples were all biologically independent. Data are expressed as means ± standard deviation; *P < 0.05.

To analyze the influence of abnormal lipid metabolism on the visual system, we developed HFD model mice using BALB/c mice. Regular intake of an HFD had already caused obesity at 1 month (Fig. 1a), and the bodyweight was increased by 114% compared with that in normal diet (ND)-fed control mice at the end of 3 months (ND mice, 30.1 ± 3.4 g; HFD mice, 34.2 ± 4.1 g, *p* < 0.05). Blood sugar levels were higher in HFD mice than in ND mice at month 3 (ND mice, 84.2 ± 13.7 mg/dL; HFD, 110.0 ± 15.6 mg/dL, *p* < 0.05) (Supplementary Fig. 1); however, these levels were not high enough to be classified as diabetic (blood sugar levels > 250 mg/dL; diabetic)^39,46,47^, and the plasma insulin levels were not changed (Supplementary Fig. 1), which is consistent with a previous study that BALB/c mice have less deterioration in glucose metabolism and insulin action by HFD^48^. There was a significant but subtle increase in blood pressure (ND mice, 119 ± 4 mmHg; HFD mice, 130 ± 12 mmHg, *p* < 0.05) (Supplementary Fig. 1), consistent with the hypothesis that systemic RAS can be induced by HFD. The mRNA levels of angiotensinogen, which is a substrate of angiotensin II, a ligand in RAS, was locally induced in the neural retina and RPE–choroid complex tissue of HFD mice, suggesting that tissue RAS could be also induced by HFD, while AT1R, a receptor in RAS, was upregulated only in the RPE–choroid (Fig. 1b). The choroid is a vascular-rich connective tissue and cannot easily be separated from the RPE; thus, analyses were conducted using complex samples.

Next, we measured visual function using electroretinography (ERG) with or without treatments with an ARB, valsartan, for the last 7 days of each 1 and 3-month study (Fig. 1c). The ARB suppressed HFD-related increase in blood pressure; however, the levels were within normal ranges (Supplementary Fig. 1). There were no significant changes in creatinine and insulin levels with or without ARB treatment in HFD mice (Supplementary Fig. 1), and no adverse events such as infections.

The a-wave amplitudes representing photoreceptor cell function were reduced at 1 month of daily HFD intake (Fig. 1d). This was not the case in HFD mice treated with ARB for 7 days at the end of the month of HFD intake. The ARB preserved a-wave amplitudes at stimulus intensities of −0.6 and 0.4 log cd-s/m^2^, indicating that HFD-induced photoreceptor dysfunction occurred via the AT1R. Significantly decreased a-wave amplitudes apparent at 3 months in HFD mice were also suppressed by ARB treatment for the last 7 days of the 3 months (Fig. 1c, e). Taken together, HFD mice already had decreased visual function at 1 month (Fig. 1d); however, visual function was preserved by 7 days of ARB administration in the 3-month study, indicating that the once-impaired visual function was recovered by ARB (Fig. 1e). Decreases in the b-wave amplitudes representing the subsequent visual response to photoreceptor cells were not significant, although there was a decreasing trend in HFD mice. The implicit times of the ERG were not changed by HFD when compared with ND mice. Administration of ARB did not alter the ERG data in ND mice. The oscillatory potentials which are suppressed under diabetic conditions^39,46,49^ were not changed in the four groups (Supplementary Fig. 2). Thus, constant intake of HFD-induced visual function impairment, which was not only avoided but recovered via AT1R blockade.

### Retinal histological changes due to HFD were remediated by AT1R blockade

**Fig. 2:**
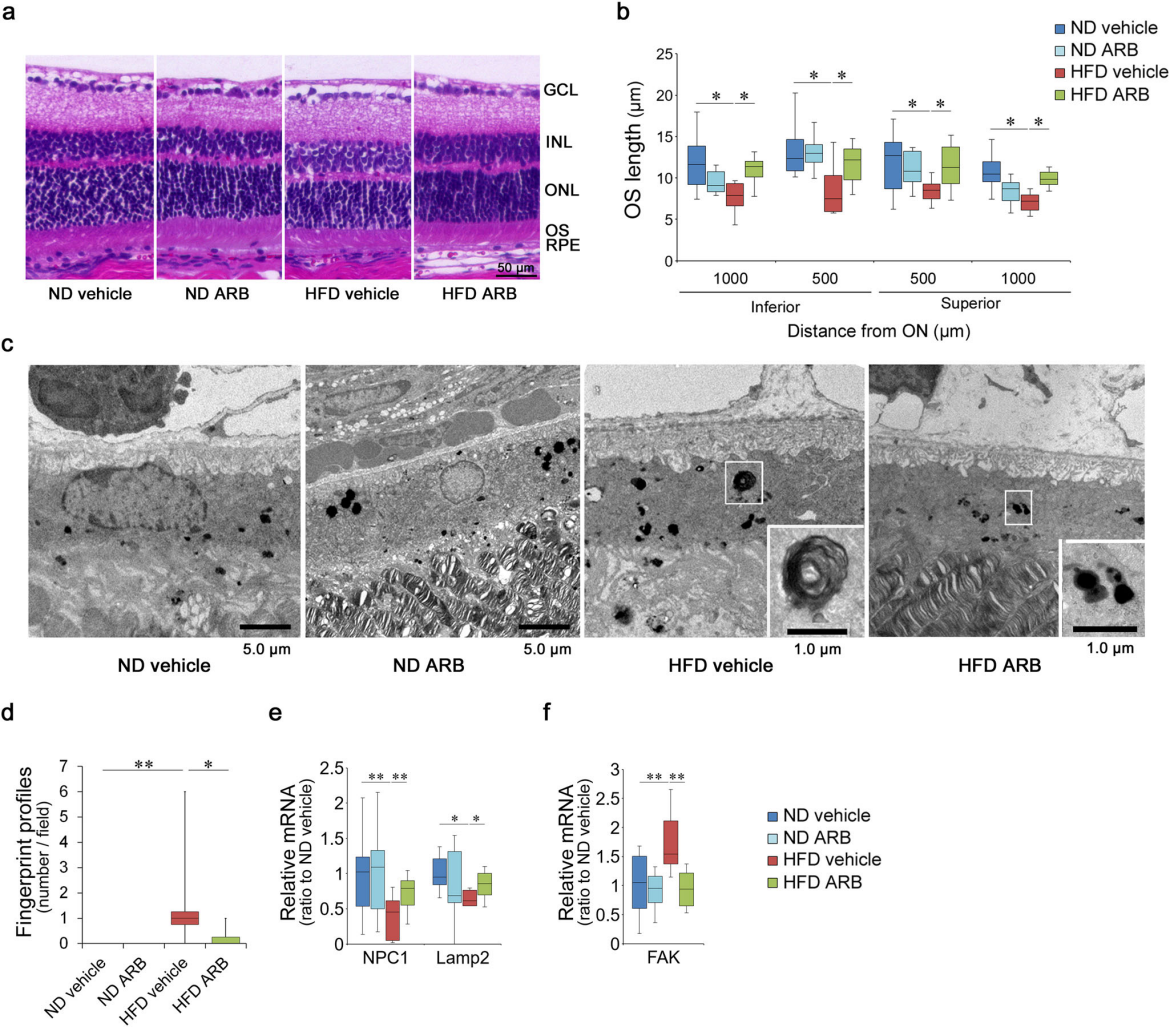
Retinal histological changes due to HFD were reversed by AT1R blockade. **a** HE staining of the retina at 1 month. **b** OS length was reduced by the HFD; however, it was recovered with AT1R blockade by ARB. **c**, **d** Electron microscopy (EM) of the RPE. Abnormal structures known as fingerprint profiles (inset in HFD vehicle sample; magnified image) generated by lysosomal deficiency appeared in the RPE of HFD mice; however, these profiles were substantially reduced after the AT1R blockade. Note that the fingerprint profiles differed from the homogenous pigment found in all mice (inset in HFD ARB sample; magnified image). **e**, **f** mRNA levels in the RPE–choroid complex. Lysosome markers, NPC1, and LAMP2 (**e**), were downregulated, and an autophagy marker, FAK (**f**), was upregulated in HFD mice, all of which were rescued by ARB. ND normal diet, HFD high-fat diet, AT1R angiotensin II type 1 receptor, ARB AT1R blocker, OS outer segment, RPE, retinal pigment epithelium. Respective numbers for ND mice treated with control, ND mice treated with ARB, HFD mice treated with control, and HFD mice treated with ARB were (**b**) *n* = 10 for each group. **d** *n* = 12 for each group; **e** 14, 14, 9, 9; **f** 14, 14, 8, 8. The samples were all biologically independent. Data are expressed as means ± standard deviation; **b** *P < 0.05 for comparison between ND and HFD both treated with vehicle; ^†^*P* < 0.05 for comparison between HFD treated with vehicle and HFD treated with ARB; **d**–**f** *P < 0.05, **P < 0.01. Scale bar, 2.0 μm (**a**), 1.0 or 5.0 μm (**b**, **c**).

We performed a histological analysis to elucidate the mechanism underlying the photoreceptor disorder induced by HFD, with or without ARB treatment, for 7 days at the end of 1-month HFD intake (Fig. 2a). We found that the photoreceptor OS length was reduced in HFD mice; however, ARB administration preserved the length (Fig. 2a, b).

RPE is essential for photoreceptor OS renewal and sustaining visual function^17,50^. In the RPE of HFD mice, abnormal structures known as fingerprint profiles were frequently observed by electron microscopy (EM) at 1 month (Fig. 2c). This structure was first reported as undigested OSs in the RPE of mice with knockout (KO) of cathepsin D, the lysosomal enzyme of the RPE^51,52^, suggesting that HFD mice exhibited lysosomal deficiency that resulted in the accumulation of undigested OSs in the RPE. Similar results were obtained in a previous study of the RPE of a postmortem eye with AMD^53^. Following ARB treatment, these structures were rarely found in HFD mice (Fig. 2c) and were significantly reduced (Fig. 2d). Note that the fingerprint profiles observed in the RPE of HFD mice treated with the vehicle were different from the small pigmented structures observed in the RPE of all the mice (insets in Fig. 2c). In parallel, lysosome markers, intracellular cholesterol transporter 1 (NPC1), and lysosome-associated membrane protein 2 (LAMP2) were downregulated in the RPE–choroid complex of HFD mice (Fig. 2e). NPC1 is required for cholesterol export from the lysosome and its disorder causes massive lysosomal accumulation of cholesterol^54^, and LAMP2 plays a critical role in lysosomal biogenesis and maturation of autophagosomes/phagosomes, and its deficiency accelerates the age-associated formation of basal laminar deposits, a characteristic finding in AMD^55^. However, the reductions were rescued by ARB (Fig. 2e). Focal adhesion kinase (FAK), which regulates autophagy^56^ and is essential for phagocytosis of OSs by the RPE^57^, was upregulated in HFD mice, suggesting that phagocytosis was not sufficiently performed and compensatory mechanisms were induced, although further studies are required; ARB returned the levels to normal (Fig. 2f). Taken together, HFD caused disorganization of RPE function related to lysosome and autophagy, which may be involved in the photoreceptor OS abnormality and visual impairment, although a direct effect of HFD on photoreceptors could not be excluded; however, the pathologies recovered with ARB treatment via AT1R blockade. ARB administration did not alter any data in ND mice.

### Accumulation of local oxidized low-density lipoprotein (ox-LDL) in the RPE and choroid of HFD mice was rescued by AT1R blockade

**Fig. 3:**
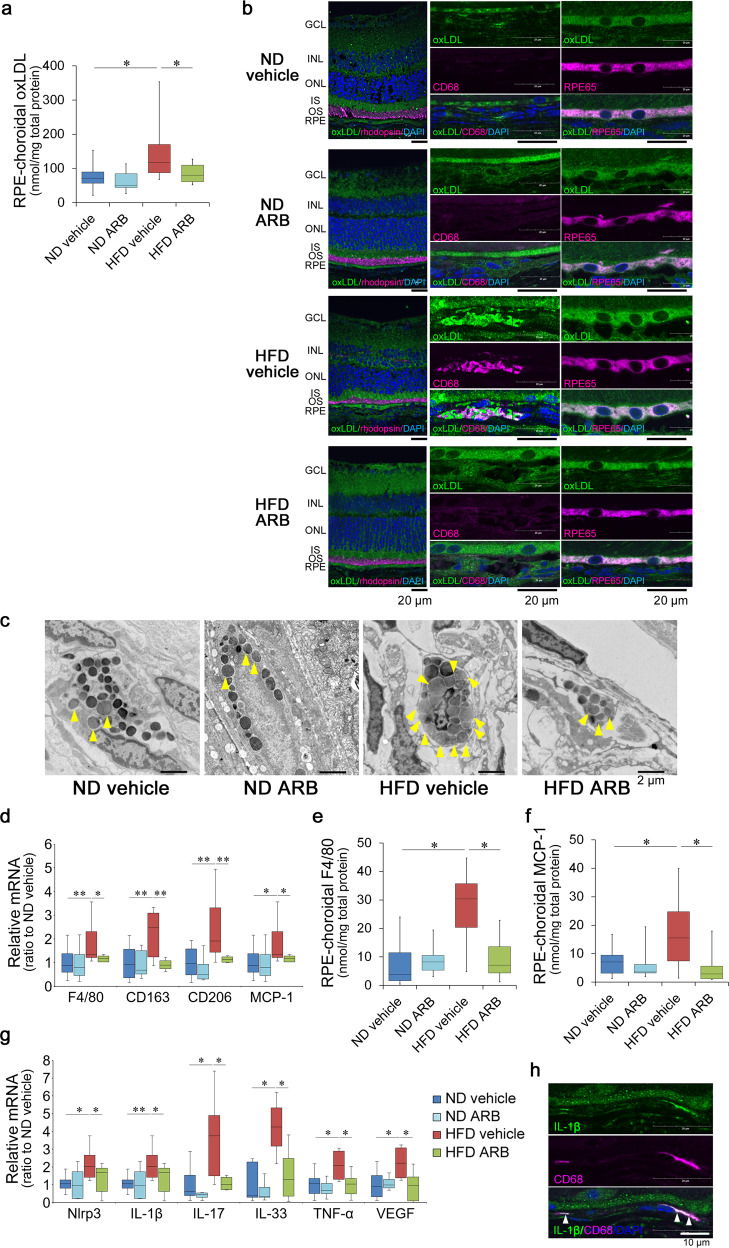
Local ox-LDL accumulation and macrophage recruitment in HFD mice were rescued by AT1R blockade. **a** ox-LDL levels in the RPE–choroid complex were measured by ELISA. The level was increased in HFD mice but rescued by AT1R blockade by the ARB. **b** Immunohistostaining showed that ox-LDL, rhodopsin (a visual pigment in the photoreceptors and concentrated in OSs), CD68 (a monocyte/macrophage marker), RPE65 (an RPE marker), and counterstaining of DAPI. ox-LDL were present in the ISs of photoreceptors, macrophages, and RPE. Note that few macrophages were found except for in the choroid of HFD mice treated with vehicle. **c** EM of macrophages in the choroid. An increased number of lipid granules (yellow arrowheads) filled the cytosol of the macrophages within the choroid of HFD mice, which was suppressed by AT1R blockade. **d** The mRNA levels of macrophage markers, F4/80, CD163, and CD206, and a recruiting factor, MCP-1; protein levels of F4/80 (**e**) and MCP-1 (**f**); and mRNA levels of inflammatory factors Nlrp3, IL-1β, IL-17, IL-33, TNFα, and VEGF (**g**) induced in the RPE–choroid of HFD mice were suppressed by the AT1R blockade. **h** IL-1β was localized in macrophages infiltrating the choroid of the HFD mice as shown by co-immunostaining with CD68 (**h**, arrowheads). ND normal diet, HFD high-fat diet, AT1R angiotensin II type 1 receptor, ARB AT1R blocker, GCL ganglion cell layer, INL inner nuclear layer, ONL outer nuclear layer, IS inner segment, OS outer segment, RPE retinal pigment epithelium. Respective numbers for ND mice treated with control, ND mice treated with ARB, HFD mice treated with control, and HFD mice treated with ARB were **a** 13, 8, 13, 13; **d**, **g** 14, 14, 8, 8; **e**, **f** 11, 8, 8, 11. The samples were all biologically independent. Data are expressed as means ± standard deviation; *P < 0.05, **P < 0.01. Scale bar, 20 μm (**b**), 2.0 μm (**c**), and 1.0 μm (**h**).

One month of regular HFD intake increased the levels of ox-LDL in the RPE–choroid complex tissue (Fig. 3a); ox-LDL is known as a clinical biomarker of metabolic syndrome^58,59^. Immunohistochemistry analysis showed that ox-LDL was found in inner segments of the photoreceptors of all groups (Fig. 3b). More importantly, it was prominently found in macrophages within the choroid, as labeled by CD68, and in the RPE of HFD mice (Fig. 3b).

Further analysis of macrophages by EM showed that the lipid granules increased and occupied the cytosol of the macrophages within the choroid of HFD mice compared with those of ND mice (Fig. 3c).

Increased cholesterol uptake may induce macrophage activation, migration, and inflammation^10,49,60^. In fact, the macrophage markers F4/80, CD163, and CD206 were upregulated in the RPE–choroid of HFD mice (Fig. 3d), suggesting that macrophages were recruited to the choroid by HFD feeding. Consistently, MCP-1, which is secreted by the RPE^23^ and involved in AMD pathogenesis by recruiting macrophages^24,25^, was increased in the RPE–choroid complex by HFD (Fig. 3d). Increased protein levels of F4/80 (Fig. 3e) and MCP-1 (Fig. 3f) were confirmed.

However, ox-LDL levels in the RPE–choroid complex tissue (Fig. 3a), lipid granules in the macrophages in the choroid (Fig. 3c, yellow arrows), and macrophage infiltration to the choroid (Fig. 3d, e) were suppressed after ARB treatment in HFD mice. In parallel, MCP-1 was suppressed by ARB, suggesting that HFD also affected the RPE to induce the cytokines through AT1R signaling, which may have promoted macrophage recruitment as one of the modulators. Alternatively or additionally, a subsequent inflammatory cycle may be involved in macrophage recruitment, as previously reported in other AMD models^6^, and increased MCP-1 levels may have been further accelerated by the recruited macrophages. In fact, a series of inflammatory cytokines related to AMD were upregulated locally in the RPE–choroid. Nlrp3 and IL-1β^61–64^, which are inflammasome-related molecules; IL-17, which mediates innate and acquired immunity related to AMD, as reported both in animals^65,66^ and humans^67–69^; IL-33, which amplifies the innate immune response and attacks the RPE^70^; TNF-α, which regulates the transcription of Nlrp3^71^; and VEGF, which can be directly associated with the development of late AMD with choroidal neovascularization^72^, were upregulated by the HFD (Fig. 3g). However, induction of this cytokine was suppressed to levels comparable with those of ND mice by systemic ARB administration (Fig. 3e–g). IL-1β was expressed in the macrophages recruited to the choroid of HFD mice as shown by co-immunostaining for IL-1β and CD68 (Fig. 3h); note that few macrophages were found in the choroid in the other groups (Fig. 3b). ARB did not affect the data of ND mice.

Therefore, the HFD recruited the ox-LDL-loaded and activated macrophages to the choroid in response to RAS activation.

### Macrophages were responsible for RPE disorder and visual impairment

**Fig. 4:**
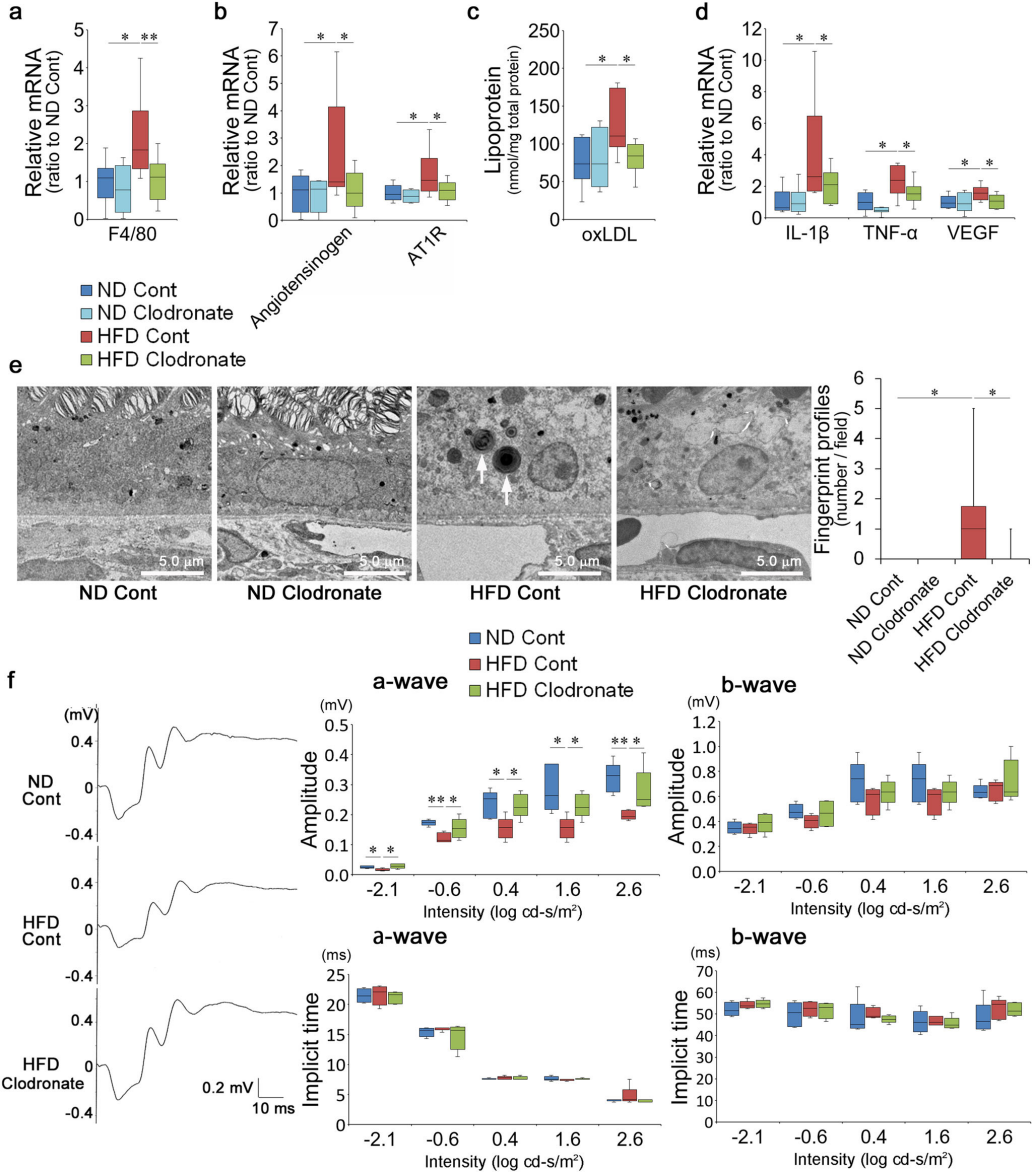
Macrophages were responsible for visual impairment and RPE disorder. **a** Macrophage elimination in HFD mice by clodronate liposomes was confirmed by decreases in F4/80 mRNA in the RPE–choroid. The control treatment was done using control liposomes. **b**–**d** Macrophage elimination repressed angiotensinogen and AT1R (**b**) and reduced ox-LDL (**c**) and the inflammatory cytokines IL-1β, TNFα, and VEGF (**d**) in the RPE–choroid. **e** EM showed that abnormal fingerprint profiles (arrows), representing lysosomal deficiency, in the RPE were rare in the absence of macrophages. Control liposomes did not cause any differences in ND mice. **f** Representative ERG waveforms from individual mice, the amplitudes, and implicit times of a- and b-waves at 1 month. Visual function was preserved in HFD mice when macrophages were eliminated. HFD high-fat diet, RPE retinal pigment epithelium, AT1R angiotensin II type 1 receptor, EM electron microscopy, ERG electroretinogram. Respective numbers for ND mice treated with control, ND mice treated with clodronate, HFD mice treated with control, and HFD mice treated with clodronate were **a**, **c** 10, 8, 10, 10; **b**, **d** 6, 8, 10, 10; **e** *n* = 10 for each group; **f** *n* = 5 for each group. The samples were all biologically independent. Data were expressed as means ± standard deviation; *P < 0.05, **P < 0.01. Scale bar, 5.0 μm.

To assess the impact of macrophage activation on visual impairment and the RPE disorder, we eliminated activated macrophages by intraperitoneal injection of clodronate liposomes in HFD mice at 1 month. Elimination was confirmed by downregulation of F4/80 in the RPE–choroid complex samples to levels comparable to those in ND mice (Fig. 4a). Under the same conditions, angiotensinogen and AT1R were repressed (Fig. 4b), ox-LDL was reduced (Fig. 4c), and local levels of IL-1β, TNF-α, and VEGF were all suppressed (Fig. 4d) in the RPE–choroid. Thus, the major source of local RAS activity, ox-LDL, and cytokine induction in HFD mice were the macrophages. In addition, the fingerprint profiles, representing undigested deposits due to lysosome deficiency^51,52^, in the RPE were rarely found (Fig. 4e). There were no changes in the ND mice treated with clodronate liposomes. Visual impairment due to HFD, depicted as a reduction in the a-wave amplitude, was rescued by macrophage elimination (Fig. 4f).

Therefore, macrophages loaded with excessive ox-LDL and activated and recruited to the choroid were responsible for RPE pathogenesis and visual disorders related to the HFD.

### Accumulation of ox-LDL in macrophages of HFD mice occurred through AT1R signaling

**Fig. 5:**
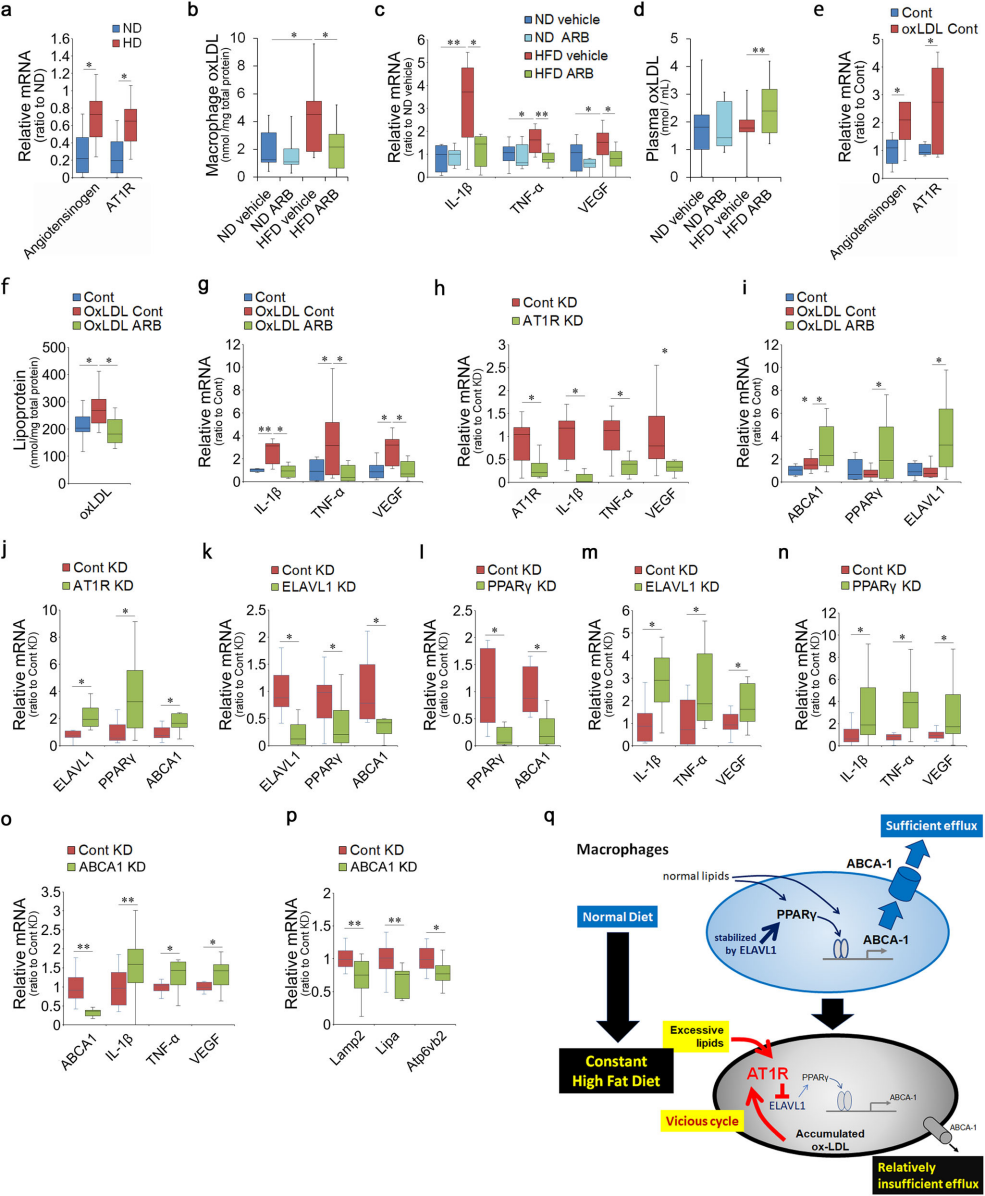
Macrophage ox-LDL in HFD mice accumulated through the AT1R/ELAVL1/PPARγ/ABCA1 axis. **a**–**d** Peritoneal macrophages derived from ND or HFD mice with or without ARB administration. **a** Peritoneal macrophages in the HFD mice showed upregulation of angiotensinogen and AT1R. **b**, **c** Levels of ox-LDL (**b**) and mRNAs of the inflammatory cytokines IL-1β, TNFα, and VEGF (**c**) were increased in macrophages from HFD mice. However, the levels were suppressed by systemic ARB administration. **d** The levels of ox-LDL in the plasma were not changed by HFD and were increased via AT1R blockade. **e**–**p** Peritoneal macrophages from ND mice were cultured with or without ox-LDL. **e** Adding ox-LDL to the culture-induced angiotensinogen and AT1R mRNAs. **f**, **g** ox-LDL loading increased ox-LDL levels (**f**) and mRNAs of the inflammatory cytokines IL-1β, TNFα, and VEGF (**g**) in the macrophages, which was suppressed by ARB. **h** In macrophages cultured without ox-LDL, AT1R KD repressed the cytokines. **i** ox-LDL loading induced ABCA1, and AT1R blockade further upregulated ABCA1. PPARγ and ELAVL1 were unchanged by ox-LDL and were upregulated by AT1R blockade. **j** AT1R KD upregulated ELAVL1, PPARγ, and ABCA1; **k** ELAVL1 KD repressed PPARγ and ABCA1, and **l** PPARγ KD repressed ABCA1. Under ELAVL1 KD (**m**), PPARγ KD (**n**), and ABCA1 KD (**o**) conditions, the cytokines were upregulated. **p** ABCA1 KD suppressed the lysosomal mRNAs of Lamp2, Lipa, and Atp6v1b2. **q** In the macrophages of ND mice, lipid loading induces PPARγ with the direct and/or indirect action of ELAVL1 to upregulate ABCA1 for sufficient efflux of cholesterol. However, in macrophages from HFD mice, excessive lipid loading activates AT1R, which suppresses ELAVL1, resulting in repression of PPARγ and relative suppression of ABCA1; thus, cholesterol efflux becomes insufficient. Resulting in cholesterol accumulation in the macrophages may further activate AT1R, prolonging the cycle. ND normal diet, HFD high-fat diet, AT1R angiotensin II type 1 receptor, ARB AT1R blocker. Respective numbers for ND mice treated with control, ND mice treated with ARB, HFD mice treated with control, and HFD mice treated with ARB were **a** 8 for each group; **b** 13, 8, 11, 13; **c** 10, 8, 10, 8; **d** 10, 8, 14, 14. **e** Respective numbers for control and ox-LDL + control were 8 and 11. **f** *n* = 8 for each group; **h** *n* = 12 for each group. **i** Respective numbers for control, ox-LDL, and oxLDL + ARB was 8, 10, 9. **j**–**p** *n* = 12 for each group. The samples were all biologically independent. Note that KD experiments were performed using siRNA and had no additional ox-LDL loading. Data are expressed as means ± standard deviation; *P < 0.05, **P < 0.01.

We further compared the macrophage conditions derived from the mouse models with or without HFD intake for 1 month and ARB treatment. We found that angiotensinogen and AT1R were induced in the peritoneal macrophages of HFD mice (Fig. 5a). Moreover, ox-LDL levels were significantly increased (Fig. 5b). However, the ox-LDL levels were suppressed by systemic AT1R blockade (Fig. 5b). The inflammatory cytokines IL-1β, TNF-α, and VEGF were upregulated by the HFD; however, this induction was suppressed by AT1R blockade (Fig. 5c). Therefore, HFD-induced ox-LDL accumulation in the macrophages occurred through the RAS.

The ox-LDL levels in the plasma were not changed by HFD alone; however, they were increased by ARB (Fig. 5d), suggesting that HFD increased total ox-LDL levels in the body and ARB prohibited ox-LDL loading in the cells so that it was sufficiently excreted into the plasma. The ARB did not cause changes in the macrophages of ND mice.

### AT1R signaling impeded the ELAV-like RNA binding protein 1 (ELAVL1)/PPARγ/ABCA1 axis and promoted ox-LDL accumulation

To assess the impact of AT1R signaling on the ox-LDL accumulation in macrophages, the primary culture of peritoneal macrophages derived from ND mice was analyzed. Under conditions of ox-LDL loading, angiotensinogen and AT1R were induced (Fig. 5e). The ox-LDL contained in the macrophages was increased; however, adding ARB to the culture reduced ox-LDL to levels comparable to those in controls with no ox-LDL loading (Fig. 5f). The inflammatory cytokines IL-1β, TNF-α, and VEGF were induced by ox-LDL in the culture, indicating that ox-LDL induced macrophage activation (Fig. 5g). The levels of all these cytokines were suppressed by ARB (Fig. 5g), and the influence of AT1R on cytokine induction was confirmed via AT1R knockdown (KD) by siRNA (Fig. 5h). These results indicate that ox-LDL-mediated AT1R signaling activated the macrophages to induce inflammatory cytokines.

Next, the mechanism of AT1R signaling and resulting effects on lipid metabolism were evaluated. Following ox-LDL loading, a cholesterol transporter, ABCA1, was induced (Fig. 5i), and AT1R blockade further upregulated ABCA1. Because ox-LDL accumulated without AT1R blockade (Fig. 5f), the ABCA1 levels induced by ox-LDL were not sufficient to excrete the loaded cholesterol.

We further analyzed the molecules that may be related to ABCA1 transcription by referring to the previous studies in other contexts. Along with this, we found that a nuclear receptor related to lipid metabolism, PPARγ, an mRNA-binding protein involved in various inflammatory pathogenesis^73^, ELAVL1, remained unchanged by ox-LDL loading; however, they were upregulated by AT1R blockade (Fig. 5i). Using siRNA-mediated KD, AT1R signaling was found to inhibit ELAVL1, PPARγ, and ABCA1 (Fig. 5j). Moreover, the induction of PPARγ and ABCA1 by ELAVL1 (Fig. 5k), and ABCA1 by PPARγ (Fig. 5l) was significant. Thus, the molecular pathway that AT1R suppressed via the ELAVL1/PPARγ/ABCA1 axis was confirmed by genetic manipulation.

We also confirmed that respective ELAVL1 and PPARγ deficiencies induce inflammatory cytokines similarly to ABCA1 deficiency. ELAVL1 KD (Fig. 5m) and PPARγ KD (Fig. 5n) induced the inflammatory cytokines IL-1β, TNFα, and VEGF and activated macrophages. ABCA1 KD increased cytokine levels (Fig. 5o) and decreased lysosome-related molecules, such as LAMP-2, Lipa, and Atp6v1b2 (Fig. 5p) in the macrophages, indicating disorganized macrophage function.

These results suggest that excessive lipid loading activated AT1R signaling, suppressed additional ELAVL1 induction, and reduced PPARγ mRNA levels, which impeded further ABCA1 induction that was sufficient for excreting overloaded cholesterol (Fig. 5q). As a result, AT1R-mediated metabolic decompensation of cholesterol disorganized the macrophage functions.

### Chronic HFD intake suppressed the ELAVL1/PPARγ/ABCA1 axis through AT1R signaling in the macrophages

**Fig. 6:**
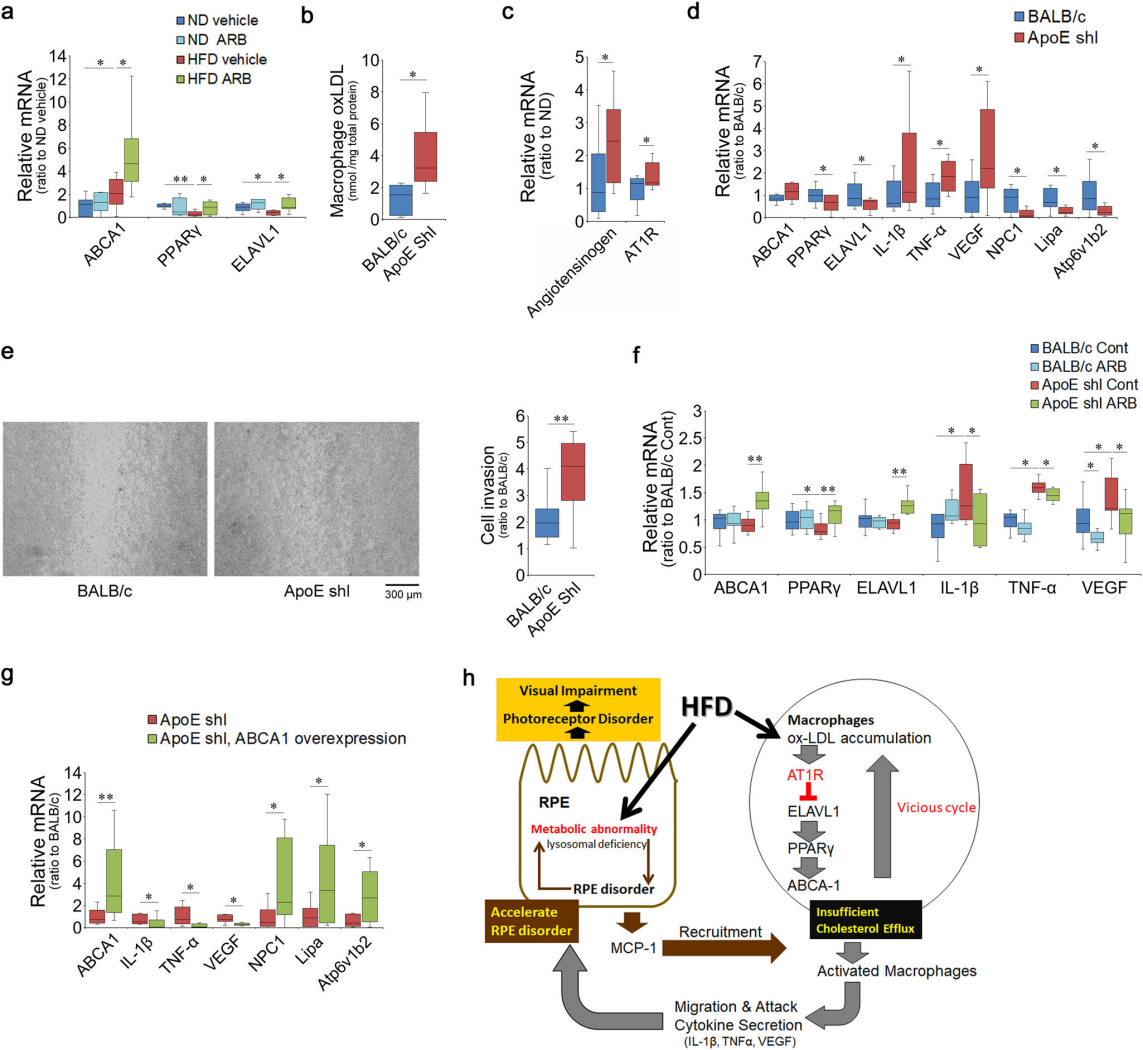
Chronic HFD loading suppressed the ELAVL1/PPARγ/ABCA1 axis through AT1R signaling in macrophages. **a** Peritoneal macrophages derived from ND or HFD mice at 1 month, with or without ARB treatment. ABCA1 was induced by HFD, and the level was further increased by AT1R blockade. HFD repressed PPARγ and ELAVL1; however, these levels were recovered by AT1R blockade. **b**–**e** Peritoneal macrophages derived from *ApoE*-deficient mice were analyzed. ox-LDL levels were increased (**b**), angiotensinogen and AT1R were upregulated (**c**), and PPARγ and ELAVL1 were downregulated, whereas the inflammatory cytokines, IL-1β, TNFα, and VEGF were induced, and lysosomal markers NPC-1, Lipa, and Atp6V1b2 were repressed (**d**) in macrophages from *ApoE*-deficient mice. **e** Scratch assay showed that peritoneal macrophages derived from *ApoE*-deficient mice migrated more frequently and extensively. **f** Peritoneal macrophages derived from *ApoE*-deficient mice and cultured with ARB showed increased levels of ABCA1, PPARγ, and ELAVL1, and decreased levels of inflammatory cytokines in the presence of ox-LDL. **g** Overexpression of ABCA1 in peritoneal macrophages derived from *ApoE*-deficient mice. ABCA1 was upregulated by the overexpression, inflammatory cytokines, IL-1β, TNFα, and VEGF were downregulated, and lysosome lysosomal markers NPC-1, Lipa, and Atp6V1b2 were upregulated. **h** Chronic HFD may affect both the RPE to induce MCP-1 which recruits macrophages, and macrophages to impair cholesterol metabolism through AT1R signaling which results in macrophage activation. The changes may progress gradually and concurrently. The activated and recruited macrophages secrete inflammatory cytokines and cause further RPE disorders including lysosome deficiency to affect visual function. ND normal diet, HFD high-fat diet, AT1R angiotensin II type 1 receptor, ARB AT1R blocker. **a** Respective numbers for ND mice treated with control, ND mice treated with ARB, HFD mice treated with control, and HFD mice treated with ARB were 10, 8, 10, 8. **b** *n* = 8, **c**, **d** *n* = 9, and **e** *n* = 14 for each group. **f** Respective numbers for BALB/c mice treated with control, BALB/c mice treated with ARB, *ApoE shl* mice treated with control, and *ApoE shl* mice treated with ARB were 12, 12, 12, 11. **g** *n* = 12 for each group. The samples were all biologically independent. Data are expressed as means ± standard deviation; *P < 0.05, **P < 0.01.

To evaluate whether the in vitro mechanism was also applicable in vivo, peritoneal macrophages derived from HFD-fed mice at 1 month, with or without ARB treatment, were analyzed. ABCA1 was induced by an HFD and further upregulated by AT1R blockade (Fig. 6a). In contrast to the in vitro results, PPARγ and ELAVL1 were suppressed by regular HFD intake (Fig. 6a); however, the levels were upregulated by the AT1R blocker to levels comparable with those in ND mice. These results suggest that ELAVL1 was suppressed in vivo through AT1R signaling in the macrophages of the mice with the chronic intake of HFD, rather than remaining unchanged as shown in in vitro analyses (Fig. 5i), and subsequently, PPARγ mRNA was downregulated. This discrepancy between the in vivo and in vitro results (Fig. 5i) may have occurred because the in vitro analyses were performed only 24 h after ox-LDL loading, whereas in vivo analyses were performed after continuous intake of various types of lipids. There were no differences by ARB administration in peritoneal macrophages derived from ND mice.

We also analyzed peritoneal macrophages of spontaneous *ApoE*-deficient mice (C.KOR/StmSlc-*Apoe*^*shl*^), another model of lipid abnormality. *ApoE* SNPs are risk factors for AMD^28–30^ and Alzheimer’s disease^74^. *ApoE* deficiency causes not only hyperlipidemia and atherosclerosis^75^ but also pathological thickening of Bruch’s membrane, which is composed of the basement membrane of the RPE in mice^76^. In the peritoneal macrophages of *ApoE*-deficient mice, ox-LDL was increased compared with that in control mice (Fig. 6b), and both angiotensinogen and AT1R were upregulated (Fig. 6c). ABCA1 was not upregulated, which likely caused ox-LDL accumulation. PPARγ and ELAVL1 were repressed (Fig. 6d), and the inflammatory cytokines IL-1β, TNF-α, and VEGF were upregulated (Fig. 6d). The lysosomal markers, NPC-1, Lipa, and ATP6v1b2, were downregulated (Fig. 6d). Thus, *ApoE* deficiency-induced similar pathogenesis in macrophages as in HFD mice. Scratch assay showed that peritoneal macrophages derived from *ApoE*-deficient mice migrated more extensively than control macrophages (Fig. 6e).

Peritoneal macrophages from *ApoE*-deficient mice cultured with ARB showed increased levels of ABCA1, PPARγ, and ELAVL1 and decreased levels of inflammatory cytokines (Fig. 6f), suggesting that AT1R blockade regulates lipid metabolism in the macrophages of *ApoE*-deficient mice. ARB did not affect macrophages derived from control mice.

Then, peritoneal macrophages derived from *ApoE*-deficient mice were transfected with the ABCA1 construct. Under ABCA1 overexpressed condition (Fig. 6g), inflammatory cytokines, IL-1β, TNFα, and VEGF were downregulated (Fig. 6g) and lysosomal markers, NPC-1, Lipa, and ATP6v1b2, were upregulated (Fig. 6g), suggesting that ABCA1 levels were critical in determining macrophage pathogenesis. These results suggest that the RAS mechanism in macrophage cholesterol efflux is applicable to other lipid metabolism abnormalities.

## Discussion

We demonstrated that HFD impaired visual function corresponding to RPE disorder, which was mainly induced by activated and locally recruited macrophages. Macrophage activation and infiltration were caused by daily HFD-mediated overloaded ox-LDL. Importantly, the molecular mechanisms of ox-LDL accumulation in the macrophages occurred through AT1R signaling and its downstream interference of the ELAVL1/PPARγ/ABCA1 axis, resulting in disorganized cholesterol efflux. Macrophages from *ApoE*-deficient mice also possessed a similar system for abnormal lipid metabolism through AT1R.

AMD is characterized by accumulated debris due to RPE disorder^77–79^. It arises as a result of abnormal metabolism of photoreceptor OSs, which are composed of lipid bilayers and phagocytosed by RPE during the visual cycle. The HFD mice in the current study developed undigested lysosomal deposits in the RPE, similar to the RPE of lysosome-deficient mice^51,52^. Reduction in the expression of lysosome markers, NPC1 and LAMP2, and abnormal expression of an autophagy marker, FAK1, supported the presence of lysosome disorganization in HFD mice. NLRP3 inflammasomes resulting in RPE damage and death^63^ and MCP-1 induction for macrophage activation and recruitment^24,25^ have been reported as fundamental for AMD pathogenesis. Photoreceptor OS shortening was consistent with results obtained in human AMD-fellow eyes that may comprise AMD in the near future^80^. Therefore, we demonstrated that HFD mice reproduced various aspects of AMD. The absence of definitive AMD models is one issue impeding AMD research, and the current model may be useful for further analyzing AMD.

Visual impairment at 1 and 3 months of daily HFD intake was restored by AT1R blockade at similar levels to those of control ND mice, suggesting that this condition during the early stages was reversible and that treatment intervention produced a significant impact. However, if this condition remains untreated, lysosomal dysfunction eventually causes RPE death through prolonged inflammasome activation^81,82^ and mitochondrial damage^77,79,83^. RPE disorder is often caused by abnormal lipid metabolism, as shown in postmortem human eyes^84,85^. A progressive hereditary disease, Stargardt disease, is caused by a mutation in *ABCA4*, another lipid transporter, which causes blindness due to lipid deposits, lipofuscin, in the RPE^86^.

Because the elimination of activated macrophages achieved suppression of the RPE disorder and visual impairment, similarly to ARB treatment, the ocular phenotypes were mainly caused by activation and infiltration of macrophages into the choroid through AT1R activation. Thus, the macrophages were more relevant in causing abnormal lysosome function in the RPE than lipid overloading to the RPE. However, the trigger of the macrophage recruitment could be related to the HFD-induced abnormal lipid metabolism in the RPE (Fig. 6h). The well-accepted concept of the pathogenesis of AMD development is based on the aging of the RPE and related metabolic decompensation, e.g. during daily digestion of lipid bilayer of photoreceptor OSs^87^. In addition, previous animal experiments estimated that RPE disorders and related macrophage recruitment are promoted by oxidative stress^25,88,89^ and/or inflammation^6,8,40,90,91^ induced in the retina and the RPE in response to light exposure and/or other stimuli. HFD may have changed lipid components of the retina and caused stress stimuli in the RPE to express macrophage inducing cytokines, such as MCP-1. Macrophages activated and recruited to the choroid by HFD in parallel to the RPE changes attacked the RPE to further facilitate the RPE–macrophage interaction to accelerate RPE disorders (Fig. 6h). These pathogeneses would gradually progress inapparently and subclinically, and when changes are accumulated, visible lesions such as drusen appear, establishing early AMD lesions. During this course, photoreceptor changes and visual function impairment also gradually progress. The current models represent the subclinical stage of the RPE changes in terms of histology, although the visual function was already impaired as recorded by ERG.

Activation of AT1R may occur through autocrine/paracrine-mediated ligand stimulation by angiotensin II generated from angiotensinogen. In addition, AT1R can be stimulated by mechanical stress^92^, which can be suppressed by the inverse agonist function of the AT1R blocker used in the current study^93–97^. The HFD was reported to alter the composition of the lipid bilayer;^98^ thus, the stability of AT1R^99^ may have been affected, indicating that mechanical stress-induced activation is involved. Otherwise, the agonistic autoantibody can be generated to activate AT1R^97^, although, this mechanism cannot be involved in vitro analyses.

We found that AT1R signaling directly reduced ELAVL1 transcription. In addition, daily in vivo HFD stimuli repressed ELAVL1 transcription in macrophages (Fig. 5q). The chronic effect of ELAVL1 repression may have reduced PPARγ mRNA directly and/or indirectly^100,101^ to impede sufficient ABCA1 expression and ox-LDL efflux. ABCA1 was upregulated in response to HFD most likely through pathways other than PPARγ^102^. However, the levels of ABCA1 were not sufficient to cause excretion of excessive lipids loaded by the HFD, and decompensation eventually induced pathogenesis. Accumulated ox-LDL in the macrophages may have further influenced the ELAVL1/PPARγ/ABCA1 axis to cause a vicious cycle. The axis was also affected in macrophages derived from *ApoE*-deficient mice and may be regulated by AT1R signaling. The relevance of ABCA1 in macrophage activation was confirmed by the overexpression of ABCA1 in the macrophages derived from *ApoE*-deficient mice. Therefore, ELAVL1 and PPARγ, as well as their upstream molecule AT1R, which can regulate ABCA1 expression and lipid metabolisms, are potential therapeutic targets for AMD and other macrophage-related pathogeneses.

ELAVL1 binds to the AU-rich element in the 3′-untranslated region of certain mRNAs to stabilize mRNAs that encode early response genes or growth-related genes, such as proto-oncogenes and growth factors^103^. Previous studies reported that ELAVL1 affects the prognosis of cancers^104^, cardiovasculopathies^105^, and autoimmune diseases^106^ by modulating cytokine and chemokine expression. Thus, ELAVL1 modulators may constitute a therapeutic approach in these fields. However, the upstream regulatory signals involved in ELAVL1 transcription are not well-understood. The finding that AT1R was upstream of ELAVL1 provides a foundation for further studies in the abovementioned fields.

A previous study reported that an SNP of angiotensin I-converting enzyme, a component of RAS and key molecule in angiotensin II production, reduces AMD risk^107^, supporting the results of our current study. Given that RAS involves a series of molecules, SNPs in RAS components, with or without combinations of the components, should be further assessed to determine the impact of RAS on AMD pathogenesis; this information is becoming more critical in the modern world, in which human diets are rich in fat. Macrophage intervention in the early stages of the disease before or around the onset of early AMD may be effective for retinal neuroprotection and recovery. ARBs are already used on a long-term basis as antihypertensive treatments; therefore, these drugs may be suitable for long-term practical treatment for preventing AMD progression. Future clinical studies analyzing the effects of ARBs on macrophages and retinal conditions are needed. In addition, it is important to elucidate the role of AT1R signaling within pathogenic macrophages in other illnesses to establish preventive therapies covering various aging diseases. In conclusion, AT1R signaling may be utilized as a future therapeutic target for aging diseases that involve macrophage disorganization, such as AMD.

## Methods

### Ethics

All animal experiments followed the ARRIVE guidelines and were conducted in accordance with the Association for Research in Vision and Ophthalmology Statement for the Use of Animals in Ophthalmic and Vision Research and the guidelines of the Animal Care Committee of Keio University (Approval No. 08002).

### Animals

Five-week-old BALB/c male mice (purchased from CLEA Japan, Tokyo, Japan) were housed in an air-conditioned room under a 12-h dark/light cycle, with *ad libitum* access to either an ND (containing 4.6% fat) or HFD containing 32% fat (CLEA Japan, Tokyo, Japan) and tap water, from 5 weeks of age for 1 or 3 months according to the experiments. In contrast to C57BL/6J mice which have a variant *RPE65* sequence and thus slow the kinetics of rhodopsin metabolism, resulting in low risk of retinal damage related to RPE disorder, BALB/c mice have a normal *RPE65* sequence and kinetics which causes retinal vulnerability under stress conditions, likely because of excessively activated metabolism^108^. Intravitreal injection of an ARB, valsartan (LKT Laboratories, St. Paul, MN, USA), at 5 mg/kg BW was performed daily for 7 days. Valsartan displays marked, i.e., >1000-fold, selectivity for AT1R over AT2R^109^ and lacks cross-reactivity with other G-protein-coupled receptors^110^. Some ARBs, notable telmisartan, have independent effects on AT1R which are mediated by PPARγ; however, PPARγ does not interact with valsartan^110,111^. The dose of ARB was optimized based on previous studies of mice^38–42^ and by analyzing ox-LDL levels; we used 5, and 20 mg/kg in the preliminary experiments, and found that the 5 mg/kg dose was sufficient to reduce ox-LDL in the RPE–choroid. Five-week-old *ApoE*-deficient male mice (background, BALB/c, C.KOR/StmSlc-*Apoe*^*shl*^, purchased from Japan SLC, Shizuoka, Japan) were fed only HFD. Casual blood sugar levels were measured with a GR-102 (TERUMO CORPORATION, Tokyo, Japan) and blood pressure was determined with an MK-2000ST (Moromachi Kikai Co., Ltd, Tokyo, Japan).

### Real-time reverse transcription-polymerase chain reaction (RT-PCR)

Total RNA was extracted from the neural retina or RPE–choroid complex sample or peritoneal macrophages with or without culture and then reverse-transcribed. Quantitative PCR analyses were performed using an ABI StepOnePlus Real-Time PCR System (Applied Biosystems, Foster City, CA, USA) in combination with SYBR system or TaqMan probes (Applied Biosystems) (Supplementary Table 1). The level of each mRNA was normalized to that of glyceraldehyde-3-phosphate dehydrogenase (GAPDH).

### Electroretinography (ERG)

Mice were dark-adapted for at least 12 h, anesthetized, and their pupils dilated with a mixture of 0.5% tropicamide and 0.5% phenylephrine (Mydrin-P, Santen Pharmaceutical, Osaka, Japan)^112^. Light pulses at each stimulus intensity were delivered through a stimulator (Ganzfeld System SG-2002; LKC Technologies, Gaithersburg, MD, USA), and electrical responses were recorded (PowerLab system 2/25; AD Instruments, NSW, Australia) and then differentially amplified and filtered through a digital bandpass filter ranging from 0.313 to 1000 Hz. The amplitude of the a-wave was measured from the baseline to the trough of the a-wave and shown as a positive value, and the b-wave was measured from the trough of the a-wave to the peak of the b-wave. Implicit times of the a- and b-waves were measured from the onset of the stimulus to the peak of each wave.

### Histology/immunohistochemistry

The mice were sacrificed, and their eyes were enucleated for further analyses. Most of the eye samples (except for the EM samples) were immediately fixed in 4% paraformaldehyde and processed into paraffin sections. The sections were stained with hematoxylin and eosin (HE) or immunostained with an ox-LDL antibody (1:200, Abcam, Cambridge, UK), rhodopsin antibody (1:10,000, Thermo Fisher Scientific, Waltham, MA, USA), CD68 antibody (1:200, Santa Cruz Biotechnology Inc., Dallas, TX, USA), RPE65 antibody (Merck Millipore, Billerica, MA, USA), and IL-1β antibody (1:200, Abcam, Cambridge, UK), and detected by Alexa 448- or 546-tagged secondary antibodies (Invitrogen, Carlsbad, CA, USA) with counterstaining by DAPI (Sigma-Aldrich, St. Louis, MO, USA). All sections were examined under a microscope equipped with a digital camera (BiorevoBZ-9000, Keyence, Osaka, Japan). The OS length was measured on both sides of the optic nerve at distances of 500 and 1000 μm using the ImageJ program (National Institutes of Health, Bethesda, MD, USA; available at http://rsb.info.nih.gov/ij/index.html).

### Electron microscopy (EM)

The mice were sacrificed, and the eyes were immediately fixed with 2.5% glutaraldehyde and post-fixed in 2% osmium tetroxide, dehydrated in a series of ethanol and propylene oxide, and embedded in epoxy resin. Sections were stained with uranyl acetate and lead citrate and examined and photographed using an electron microscope (model 1200 EXII; JEOL, Tokyo, Japan). Fingerprint profiles were counted in three images of each mouse and the data were averaged.

### Enzyme-linked immunosorbent assay (ELISA)

Protein extracts were obtained from homogenized RPE–choroid complex, peritoneal macrophages, and plasma, and proteins of interest were measured using ox-LDL (CUSABIO TECHNOLOGY LLC, Houston, TX, USA), MCP-1 (R&D Systems, Inc., Minneapolis, MN, USA) and F4/80 (LifeSpan Biosciences, Inc., Seattle, WA, USA) ELISA kits according to the manufacturer’s protocol^113^.

### Macrophage elimination by clodronate liposome

Clodronate liposome or control liposome (FormuMax Scientific Inc., Sunnyvale, CA, USA) was intraperitoneally injected at 200 μL twice at 7 days and 3 days prior to evaluation. Elimination of the macrophages was confirmed by real-time RT-PCR of F4/80.

### Peritoneal macrophages

Peritoneal macrophages were obtained by injecting 3 mL of 4% Brewer’s thioglycollate (Merck) intraperitoneally followed by a collection of the elicited peritoneal exudate cells for 4 days after injection. Exudate cells were centrifuged to use as ex vivo samples or resuspended in RPMI medium (Life Technologies, Carlsbad, CA, USA) with 100 U/mL of penicillin and 100 μg/mL of streptomycin (Nacalai Tesque, Kyoto, Japan) and 10% fetal bovine serum (Lonza, Walkersville, MD, USA) at 37 °C with 5% CO_2_. The cells were then incubated with or without 40 μg/mL of ox-LDL (Bio-Rad Laboratories, Inc., Hercules, CA, USA) for 24 h. In KD examination, AT1R, ELAVL1, PPARγ, ABCA1, and control siRNA (Thermo Fisher Scientific) were transfected with lipofectamine (Thermo Fisher Scientific) and the mRNAs of interest were analyzed by real-time PCR after 24 h. For the scratch assay, cells were grown to confluence on 24-well tissue dishes, and a single scratch was made using a sterile 1000-μl pipette tip. Photographs were taken after 12 h, and the cell-covered area was measured by Image J. In overexpression examination, ABCA1 cDNA clones (Santa Cruz Biotechnology Inc.) were transfected with UltraCruz Transfection Reagent (Santa Cruz Biotechnology Inc.) for 24 h, and then ox-LDL were added. The mRNAs of interest were analyzed by real-time PCR after 24 h.

### Statistics and reproducibility

All results are expressed as the means ± standard deviation. The values were statistically analyzed (one-way analysis of variance with Tukey’s post hoc test, or Student’s *t* test) using SPSS Statistics 25 (SPSS, Inc., Chicago, IL, USA) software, and differences were considered statistically significant at *p* < 0.05. The analyses were performed on at least duplicated technical replicates.

### Reporting summary

Further information on research design is available in the Nature Research Reporting Summary linked to this article.

## Supplementary information

Supplementary Information

Description of Additional Supplementary Files

Supplementary Data 1

Reporting Summary

## Data Availability

The datasets generated or analyzed during the current study are available from the corresponding author on reasonable request. Source data underlying plots shown in figures are provided in Supplementary Data 1.
