# Supplementary material for: Renin–angiotensin system impairs macrophage lipid metabolism to promote age-related macular degeneration in mouse models
Source: Commun Biol. 2020 Dec 9;3:767. doi: 10.1038/s42003-020-01483-2 (PMC7725839; doi:10.1038/s42003-020-01483-2)
Supplement: Supplementary file 1 — Supplementary Information [file 42003_2020_1483_MOESM1_ESM.pdf]

Renin–angiotensin system impairs macrophage lipid metabolism to promote age-related macular degeneration in mouse models

Norihiro Nagai[1,2], Hirohiko Kawashima[1,2], Eriko Toda[1], Kohei Homma[1], Hideto Osada[1], Naymel A. Guzman[1,2], Shinsuke Shibata[3], Yasuo Uchiyama[4], Hideyuki Okano[3], Kazuo Tsubota[2], Yoko Ozawa[1,2,5,6*]

[1]Laboratory of Retinal Cell Biology, Department of Ophthalmology, Keio University School of Medicine, 35 Shinanomachi, Shinjukuku, Tokyo 160-8582, Japan.

[2]Department of Ophthalmology, Keio University School of Medicine, 35 Shinanomachi, Shinjukuku, Tokyo 160-8582, Japan.

[3]Department of Physiology, Keio University School of Medicine, 35 Shinanomachi, Shinjukuku, Tokyo 160-8582, Japan.

[4]Department of Cellular and Molecular Neuropathology, Juntendo University Graduate School of Medicine, Bunkyo-Ku, Tokyo 113-0033, Japan.

[5]Department of Ophthalmology, St. Luke's International Hospital, 9-1 Akashi-cho, Chuo-ku, Tokyo 104-8560, Japan

[6]St. Luke's International University, 9-1 Akashi-cho, Chuo-ku, Tokyo 104-8560, Japan

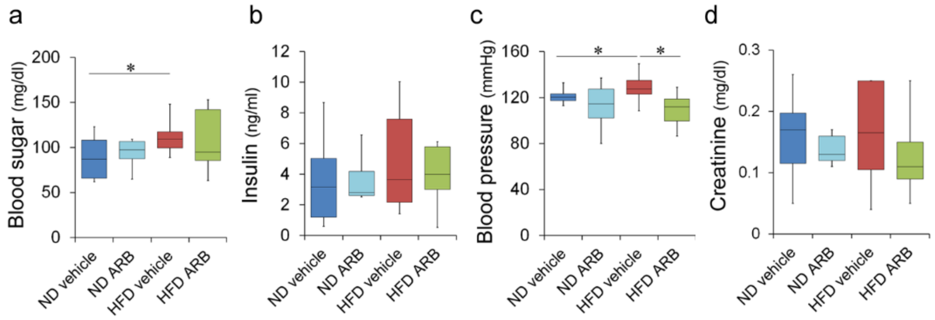

Supplementary Figure 1. Systemic data of HFD and ND mice with or without ARB.
(a) Blood sugar, (b) insulin, (c) blood pressure, and (d) creatinine levels. HFD, high fat diet; ND, normal diet; ARB, angiotensin II type 1 receptor blocker. Respective numbers for ND mice treated with control, ND mice treated with ARB, HFD mice treated with control, and HFD mice treated with ARB were (a) 17, 10, 20, 15; (b) 10, 8, 10, 10; (c) 12, 8, 9, 8; (d) n = 8 for all. The samples were all biologically independent.

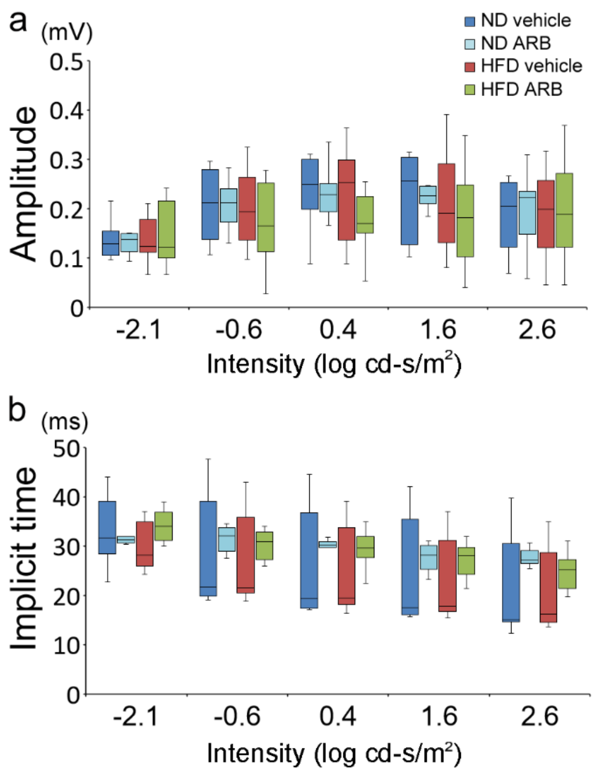

Supplementary Figure 2. Oscillatory potentials in ERG after 1 month of HFD feeding.

There were no differences between groups. ND, normal diet; HFD, high-fat diet; AT1R, angiotensin II type 1 receptor; ARB, AT1R blocker. Respective numbers for ND mice treated with control, ND mice treated with ARB, HFD mice treated with control, and HFD mice treated with ARB were 12, 8, 12, 12. The samples were all biologically independent. Data are expressed as means ± standard deviation.

Supplementary Table 1. Primers for real time RT-PCR

Genes	Forward	Reverse
ABCA1	AACAGTTTGTGGCCCTTTTG	CACAATCAGGCTGAAGACCA
Angiotensinogen	GCGGAGGCAAATCTGAACAAC A	GAAAGTGCAGCGTGCCTGAG TC
AT1R	CTGCGTCTTGTTCTGAGGTG	ACTGGTCCTTTGGTCGTGAG
Atp6v1b2	AGCCTCGTCTCACCTACAAGA	TCTCAGCGTATCTGGGAAACT T
F4/80	CTGTAACCGGATGGCAAACT	ATGGCCAAGGCAAGACATAC
ELAVL1	ACTGAACGGCTTGAGACTCC	TTCTGTGTCATGGTCCTTGG
IL-1β	AGCTCTCCACCTCAATGGAC	AGGCCACAGGTATTTTGTCG
IL-17	CAGCAGCGATCATCCCTCAAA G	CAGGACCAGGATCTCTTGCT G
IL-33	ACTGAACGGCTTGAGACTCC	TTGTGAAGGACGAAGAAGGC
Lamp2	ATGTGCCTCTCTCCGGTTAAA	GCAAGTACCCTTTGAATCTGT CA
Lipa	GGAAACAGCAGAGGAAACACC T	CACGGGAGCCAAGACTAAAA C
MCP-1	GCCTGCTGTTCACAGTTGC	TCATTGGGATCATCTTGCTG
PPARγ	AGGCCGAGAAGGAGAAGCTGT TG	TGGCCACCTCTTTGCTCTGCT G
TNF-α	GCCACCACGCTCTTCTGTCTA	GATGAGAGGGAGGCCATTTG
GAPDH	AGGAGCGAGACCCCACTAAC	GATGACCCTTTTGGCTCCAC
Nlrp3	TaqMan Gene Expression Assays, Mm00840904m1	
VEGF	TaqMan Gene Expression Assays, Mm01281449m1	
