# Supplementary material for: Renin–angiotensin system impairs macrophage lipid metabolism to promote age-related macular degeneration in mouse models
Source: Commun Biol. 2020 Dec 9;3:767. doi: 10.1038/s42003-020-01483-2 (PMC7725839; doi:10.1038/s42003-020-01483-2)
Supplement: Supplementary file 2 — Description of Additional Supplementary Files [file 42003_2020_1483_MOESM2_ESM.pdf]

Description of Additional Supplementary Files

File Name: Supplementary Data 1
Description:

Fig 1b. mRNA levels of angiotensinogen and AT1R in the retina and RPE-choroid tissue. ND, normal diet; HFD, high fat diet.

Fig 1d. Amplitudes and implicit times of a- and b- waves of ND mice treated with vehicle, ND mice treated with ARB, HFD mice treated with vehicle, and HFD mice treated with ARB (1 month).

Fig 1e. Amplitudes and implicit times of a- and b- waves of ND mice treated with vehicle, ND mice treated with ARB, HFD mice treated with vehicle, and HFD mice treated with ARB (3 months).

Fig 2b. OS length of ND mice treated with vehicle, ND mice treated with ARB, HFD mice treated with vehicle, and HFD mice treated with ARB.

Fig 2d. Number of fingerprint profiles in the retinal pigment epithelial cells of ND mice treated with vehicle, ND mice treated with ARB, HFD mice treated with vehicle, and HFD mice treated with ARB.

Fig 2e. mRNA levels of lysosome markers, NPC1 and LAMP2 in the RPE-choroid tissue of ND mice treated with vehicle, ND mice treated with ARB, HFD mice treated with vehicle, and HFD mice treated with ARB.

Fig 2f. mRNA levels of FAK in the RPE-choroid tissue of ND mice treated with vehicle, ND mice treated with ARB, HFD mice treated with vehicle, and HFD mice treated with ARB.

Fig 3a. ox-LDL levels in the RPE-choroid tissue of ND mice treated with vehicle, ND mice treated with ARB, HFD mice treated with vehicle, and HFD mice treated with ARB.

Fig 3d. mRNA levels of NPC1, LAMP2, FAK, F4/80, CD163, CD206, and MCP-1 in the RPE-choroid tissue of ND mice treated with vehicle, ND mice treated with ARB, HFD mice treated with vehicle, and HFD mice treated with ARB.

Fig 3e. F4/80 levels in the RPE-choroid tissue of ND mice treated with vehicle, ND mice treated with ARB, HFD mice treated with vehicle, and HFD mice treated with ARB.

Fig 3f. MCP-1 levels in the RPE-choroid tissue of ND mice treated with vehicle, ND mice treated with ARB, HFD mice treated with vehicle, and HFD mice treated with ARB.

Fig 3g. mRNA levels of inflammatory factors Nlrp3, IL-1β, IL-17, IL-33, TNFα, and VEGF in the RPE-choroid tissue of ND mice treated with vehicle, ND mice treated with ARB, HFD mice treated with vehicle, and HFD mice treated with ARB.

Fig 4a. mRNA levels of F4/80 in the RPE-choroid tissue of ND mice treated with control, ND mice treated with clodronate, HFD mice treated with control, and HFD mice treated with clodronate.

Fig 4b. mRNA levels of angiotensinogen and AT1R in the retina and RPE-choroid tissue of ND mice treated with control, ND mice treated with clodronate, HFD mice treated with control, and HFD mice treated with clodronate.

Fig 4c. ox-LDL levels in the RPE-choroid tissue of ND mice treated with control, ND mice treated with clodronate, HFD mice treated with control, and HFD mice treated with clodronate.

Fig 4d. mRNA levels of inflammatory factors IL-1β, TNFα, and VEGF in the RPE-choroid tissue of ND mice treated with control, ND mice treated with clodronate, HFD mice treated with control, and HFD mice treated with clodronate.

Fig 4e. Number of onion body in ND mice treated with control, ND mice treated with clodronate, HFD mice treated with control, and HFD mice treated with clodronate.

Fig 4f. Amplitudes and implicit times of a- and b- waves of ND mice treated with control, ND mice treated with clodronate, HFD mice treated with control, and HFD mice treated with clodronate.

Fig 5a. mRNA levels of angiotensinogen and AT1R in the peritoneal macrophages. ND, normal diet; HFD, high fat diet.

Fig 5b. ox-LDL levels in the peritoneal macrophages of ND mice treated with vehicle, ND mice treated with ARB, HFD mice treated with vehicle, and HFD mice treated with ARB.

Fig 5c. mRNA levels of inflammatory factors IL-1β, TNFα, and VEGF in the peritoneal macrophages of ND mice treated with vehicle, ND mice treated with ARB, HFD mice treated with vehicle, and HFD mice treated with ARB.

Fig 5d. ox-LDL levels in the plasma of ND mice treated with vehicle, ND mice treated with ARB, HFD mice treated with vehicle, and HFD mice treated with ARB.

Fig 5e. mRNA levels of angiotensinogen and AT1R in the peritoneal macrophages cultured with or without ox-LDL.

Fig 5f. mRNA levels of angiotensinogen and AT1R in the peritoneal macrophages cultured with or without ox-LDL.

Fig 5g. mRNA levels of IL-1β, TNFα, and VEGF in the peritoneal macrophages cultured with or without ox-LDL and with ARB.

Fig 5h. In macrophages cultured without ox-LDL, AT1R KD repressed mRNA of the cytokines.

Fig 5i. In macrophages, ox-LDL loading induced ABCA1 mRNA, and AT1R blockade further upregulated ABCA1 mRNA. PPARγ and ELAVL1 were unchanged by ox-LDL and were upregulated by AT1R blockade.

Fig 5j. In macrophages, AT1R KD upregulated ELAVL1, PPARγ, and ABCA1 mRNA.

Fig 5k. In macrophages, ELAVL1 KD suppressed PPARγ and ABCA1 mRNA.

Fig 5l. In macrophages, PPARγ KD suppressed ABCA1 mRNA.

Fig 5m. In macrophages, ELAVL1 KD upregulated mRNA levels of cytokines.

Fig 5n. In macrophages, PPARγ KD upregulated mRNA levels of cytokines.

Fig 5o. In macrophages, ABCA1 KD upregulated mRNA levels of cytokines.

Fig 5p. In macrophages, ABCA1 KD suppressed the lysosomal mRNAs of Lamp2, Lipa, and Atp6v1b2.

Fig 6a. Peritoneal macrophages derived from ND or HFD mice at 1 month, with or without ARB treatment. ABCA1 mRNA was induced by HFD, and the level was further increased by AT1R blockade. HFD repressed PPARγ and ELAVL1 mRNA; however, these levels were recovered by AT1R blockade.

Fig 6b. ox-LDL levels in the peritoneal macrophages of BALB/c and *ApoE*-deficient mice.

Fig 6c. mRNA levels of angiotensinogen and AT1R in the peritoneal macrophages of BALB/c and *ApoE*-deficient mice.

Fig 6d. mRNA levels of PPARγ, ELAVL1, IL-1β, TNFα, VEGF, NPC-1, Lipa, and Atp6V1b2 in the peritoneal macrophages of BALB/c and *ApoE*-deficient mice.

Fig 6e. Area of cell migration in scratch assay with peritoneal macrophages derived from BALB/c and *ApoE*-deficient mice.

Fig 6f. Peritoneal macrophages derived from *ApoE*-deficient mice and cultured with ARB showed increased mRNA levels of ABCA1, PPARγ, and ELAVL1, and decreased mRNA levels of inflammatory cytokines in the presence of ox-LDL.

Fig 6g. mRNA levels of ABCA1, inflammatory cytokines, and lysosomal markers in ABCA-1-overexpressed peritoneal macrophages derived from *ApoE*-deficient mice.

Supplementary 1. Blood sugar, insulin and creatinine levels and blood pressure of ND mice treated with vehicle, ND mice treated with ARB, HFD mice treated with vehicle, and HFD mice treated with ARB.

Supplementary 2. Amplitudes and implicit times of Op-waves of ND mice treated with vehicle, ND mice treated with ARB, HFD mice treated with vehicle, and HFD mice treated with ARB.
