# Supplementary material for: Renin–angiotensin system impairs macrophage lipid metabolism to promote age-related macular degeneration in mouse models
Source: Commun Biol. 2020 Dec 9;3:767. doi: 10.1038/s42003-020-01483-2 (PMC7725839; doi:10.1038/s42003-020-01483-2)
Supplement: Supplementary file 4 — Reporting Summary [file 42003_2020_1483_MOESM4_ESM.pdf]

nature research

Corresponding author(s): Yoko Ozawa

Last updated by author(s): Oct 15, 2020

Reporting Summary

Nature Research wishes to improve the reproducibility of the work that we publish. This form provides structure for consistency and transparency in reporting. For further information on Nature Research policies, see our Editorial Policies and the Editorial Policy Checklist.

Statistics

For all statistical analyses, confirm that the following items are present in the figure legend, table legend, main text, or Methods section.

n/a	Confirmed	
☐	☒	The exact sample size (n) for each experimental group/condition, given as a discrete number and unit of measurement
☐	☒	A statement on whether measurements were taken from distinct samples or whether the same sample was measured repeatedly
☐	☒	The statistical test(s) used AND whether they are one- or two-sided<br>*Only common tests should be described solely by name; describe more complex techniques in the Methods section.*
☐	☒	A description of all covariates tested
☐	☒	A description of any assumptions or corrections, such as tests of normality and adjustment for multiple comparisons
☐	☒	A full description of the statistical parameters including central tendency (e.g. means) or other basic estimates (e.g. regression coefficient) AND variation (e.g. standard deviation) or associated estimates of uncertainty (e.g. confidence intervals)
☐	☒	For null hypothesis testing, the test statistic (e.g. F, t, r) with confidence intervals, effect sizes, degrees of freedom and P value noted<br>*Give P values as exact values whenever suitable.*
☒	☐	For Bayesian analysis, information on the choice of priors and Markov chain Monte Carlo settings
☐	☒	For hierarchical and complex designs, identification of the appropriate level for tests and full reporting of outcomes
☐	☒	Estimates of effect sizes (e.g. Cohen's d, Pearson's r), indicating how they were calculated

Our web collection on statistics for biologists contains articles on many of the points above.

Software and code

Policy information about availability of computer code

Data collection	All results were expressed as means ± standard deviation.
Data analysis	The values were statistically analyzed (one-way ANOVA with Tukey's post hoc test, or Student's t-test) using SPSS Statistics 25 (IBM, Armonk, NY, USA) software, and differences were considered statistically significant at P < 0.05.

For manuscripts utilizing custom algorithms or software that are central to the research but not yet described in published literature, software must be made available to editors and reviewers. We strongly encourage code deposition in a community repository (e.g. GitHub). See the Nature Research guidelines for submitting code & software for further information.

Data

Policy information about availability of data

All manuscripts must include a data availability statement. This statement should provide the following information, where applicable:

- Accession codes, unique identifiers, or web links for publicly available datasets
- A list of figures that have associated raw data
- A description of any restrictions on data availability

The datasets generated or analyzed during the current study are available from the corresponding author on reasonable request.

Field-specific reporting

Life sciences study design

All studies must disclose on these points even when the disclosure is negative.

Sample size	Sample size was determined referring to previous related studies.
Data exclusions	Data were excluded in some of the real time PCR experiments, when the amplification was not appropriately obtained; there were no particular pre-established criteria.
Replication	All attempts at replication were successful.
Randomization	We randomly divided the commercially available mice into groups first, then the mice in each group were fed and treated accordingly.
Blinding	During ERG and immunohistochemistry data collection, the investigators were blinded, and regarding other experiments, the investigators were not blinded, however, the calculations were automatically performed and there were no bias from the investigators.

Reporting for specific materials, systems and methods

We require information from authors about some types of materials, experimental systems and methods used in many studies. Here, indicate whether each material, system or method listed is relevant to your study. If you are not sure if a list item applies to your research, read the appropriate section before selecting a response.

Materials & experimental systems

n/a	Involved in the study	
☐	☒	Antibodies
☒	☐	Eukaryotic cell lines
☒	☐	Palaeontology and archaeology
☐	☒	Animals and other organisms
☒	☐	Human research participants
☒	☐	Clinical data
☒	☐	Dual use research of concern

Methods

n/a	Involved in the study	
☒	☐	ChIP-seq
☒	☐	Flow cytometry
☒	☐	MRI-based neuroimaging

Antibodies

Antibodies used	ox-LDL antibody (1:200, Abcam, Cambridge, UK)<br>rhodopsin antibody (1:10000, Thermo Fisher Scientific, Waltham, MA, USA)<br>CD68 antibody (1:200, Santa Cruz Biotechnology Inc., Dallas, TX, USA),<br>RPE65 antibody (Merck Millipore, Billerica, MA, USA),<br>IL-1β antibody (1:200, Abcam, Cambridge, UK)
Validation	All antibodies were used according to the manufacturer's protocols.

Animals and other organisms

Policy information about studies involving animals; ARRIVE guidelines recommended for reporting animal research

Laboratory animals	Five-week-old BALB/c male mice (purchased from CLEA Japan, Tokyo, Japan)<br>Five-week-old ApoE-deficient male mice (background, BALB/c, C.KOR/StmSlc-Apoeshl, purchased from Japan SLC, Shizuoka, Japan)
Wild animals	n/a
Field-collected samples	n/a
Ethics oversight	All animal experiments followed the ARRIVE guidelines and were conducted in accordance with the Association for Research in Vision and Ophthalmology Statement for the Use of Animals in Ophthalmic and Vision Research and the guidelines of the Animal Care Committee of Keio University (Approval No. 08002).

Note that full information on the approval of the study protocol must also be provided in the manuscript.
